# The first complete chloroplast genome sequence of *Pentaphragma spicatum* Merr. (Pentaphragmataceae) and phylogenetic analysis

**DOI:** 10.1080/23802359.2023.2290339

**Published:** 2023-12-12

**Authors:** Zhuo Cheng, Bing-ning Yu, Xin-yi Huang, Zhen-jun Bin, Si-Zhao Liu, Zhao-jin Chi

**Affiliations:** a College of Life and Environmental Sciences, Minzu University of China, Beijing, China; b Guangxi Subtropical Crops Research Institute, Guangxi, China; c School of Ethnology and Sociology, Minzu University of China, Beijing, China

**Keywords:** Complete chloroplast genome, pentaphragmataceae, *pentaphragma spicatum* merr, phylogenetic analysis

## Abstract

*Pentaphragma spicatum* Merr. is an endemic wild edible plant of China belonging to the Pentaphragmataceae family. It is widely consumed by Shangsi County resident in Guangxi Fangchenggang. Initially, *Pentaphragma* was classified as a genus within the Campanulaceae family, but, later it was treated as part of the Pentaphragmataceae family. However, the chloroplast genome of Pentaphragmataceae has not yet been reported. In this study, we sequenced the first complete chloroplast (cp) genome of *P. spicatum* from Guangxi, China. The whole genome was 154,229 bp in length, consisting of a pair of inverted repeats (IR each 25,572 bp), a large single-copy region (LSC 84,884 bp), and a small single-copy region (SSC 18,201 bp). The complete genome contained 129 genes, including 87 protein-coding genes, 34 tRNA, and 8 rRNA genes. The overall GC content of the whole genome was 37.71%. Based on a maximum-likelihood phylogenetic analysis, it has been determined that *P. spicatum* is not phylogenetically related to Campanulaceae and supports the decision to classify it as a separate family, Pentaphragmataceae. The complete chloroplast genome of *P. spicatum* will help enhance and integrate the existing genome data of Asterales. This will provide insights into the phylogenetic relationship within Campanulaceae.

## Introduction

**Figure 1.**
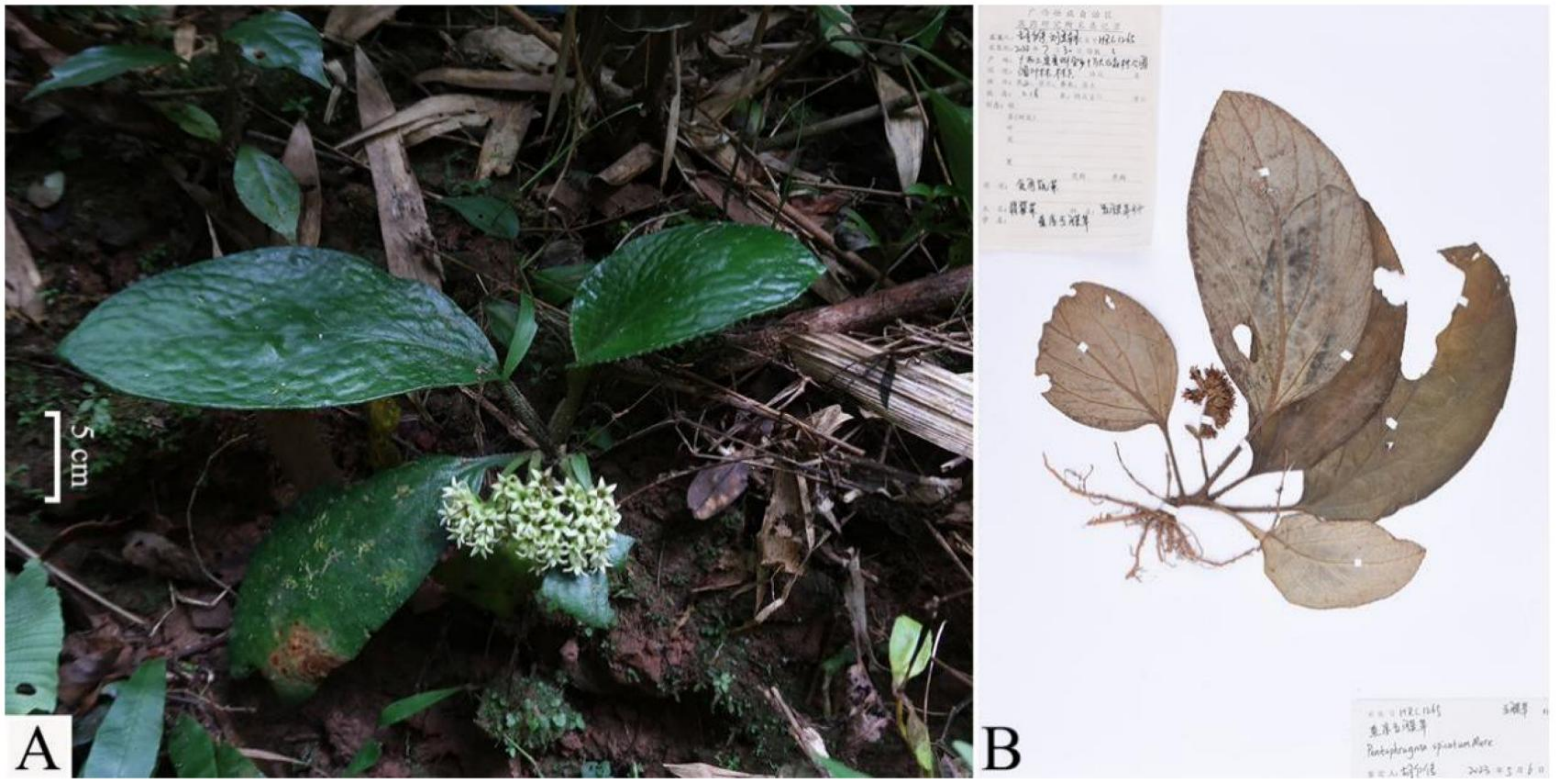
A. Plant image of *Pentaphragma spicatum*: Fleshy herb, short stem, with white or yellow-green corolla, and the sepals are shorter than the corolla. This photo was photographed by Sizhao Liu at Shangsi County in Guangxi. B. "voucher specimen of *Pentaphragma spicatum.*

*Pentaphragma spicatum* Merr., a member of the family Pentaphragmataceae, was described by Merr in 1922 (Wu and Raven 2011). This species is endemic to China and is exclusively found in the Guangxi Zhuang Autonomous Region, Guangdong Province, and Hainan Province of China (Figure 1). It is a perennial plant that grows in the subtropical biome and is currently only can be found in the wild. In Guangxi, the plant is known as "jade vegetable" by the locals due to its jade-like edible leaves. This plant can be harvested and stored for up to a month without signs of deterioration and used in stir-fried dishes or soups. Additionally, local people have found that boiling the whole plant in water and using the resulting infusion externally can be effective for treating rheumatism, bruises, promoting blood circulation, and alleviating blood stasis (Hu et al. 2023). *Pentaphragma spicatum* can be utilized as a source of domesticated species and can provide valuable genetic resources for the development of new crops through hybrid screening (Pandey et al. 2008).

*Pentaphragma* Zucc. ex Rchb. was previously classified as a genus of Campanulaceae but was later transferred to a new family, Pentaphragmataceae. Investigating the phylogenetic relationships between Pentaphragmataceae and other Asterales plants using chloroplast genome data is of significant importance. However, there is currently limited research on the chloroplast genome of Pentaphragmataceae, and no genomic studies have been conducted on *P. spicatum* in particular. Therefore, we present the first complete chloroplast genome sequence of *P. spicatum* to provide a genomic resource and clarify its phylogenetic relationship with other species in the Angiospermae family.

## Materials and methods

The total genomic DNA was extracted from dried leaves collected from Shangsi County, Fangchenggang City (Guangxi, China, E 108°13′, N 22°18′). A voucher herbarium specimen (Accession number: HRC1265; contact person: Renchuan Hu; email: hrcgxmi@163.com) was deposited at the Guangxi Institute of Traditional Medical and Pharmaceutical Sciences (http://www.cfh.ac.cn/Subsite/Default.). The total genomic DNA was extracted from the fresh leaves using the modified CTAB method (Doyle and Doyle 1987), and libraries were prepared using the TruePrep DNA Library Prep Kit (Vazyme Biotech Co., Ltd, Nanjing, CN). Genomic paired-end sequencing was conducted using the Illumina Novaseq 6000 platform, resulting in the generation of approximately 5 GB of data. The chloroplast genome was assembled and analyzed using the program NOVOPlasty-4.3.1 (Dierckxsens et al. 2017). Annotation was performed with CPGView (http://www.1kmpg.cn/cpgview/) to determine the initial location of the chloroplast genome and the IR region and to annotate the genes (Liu et al. 2023), with the chloroplast genome of *Gymnanthemum amygdalinum* (MT795180) serving as a reference. The annotations were manually proofread for errors, and the reference used was Zhou et al. (2021). The final chloroplast genome of *P. spicatum* was deposited in the NCBI GenBank under accession number: OQ942205.

Fifty-six single copy protein-coding genes (PCGs) were extracted from 26 chloroplast sequences using the PhyloSuite_v1.2.3 software (Zhang et al. 2020; Xiang et al. 2023). They were aligned using the MAFFT algorithm (Katoh et al. 2019). All these single gene alignments were concatenated to create a document for phylogenetic analyses. The best-fit model, TVM + F+R3, was determined using the Bayesian information criterion (BIC) with the ModelFinder2 program (Kalyaanamoorthy et al. 2017). To determine its phylogenetic position, a maximum likelihood (ML) tree was constructed by IQ-TREE and Bayesian inference (BI) analysis was performed with MrBayes based on the complete chloroplast genome sequences of 15 other Asterales species and six Apiales species through PhyloSuite_v1.2.3 software. Phylogenetic trees were visualized, rooted with *Lithospermum erythrorhizon*, *Trigonotis peduncularis*, and *Cordia dichotoma*, and edited using the online tool Interactive Tree of Life (https://itol.embl.de)

## Results

**Figure 2.**
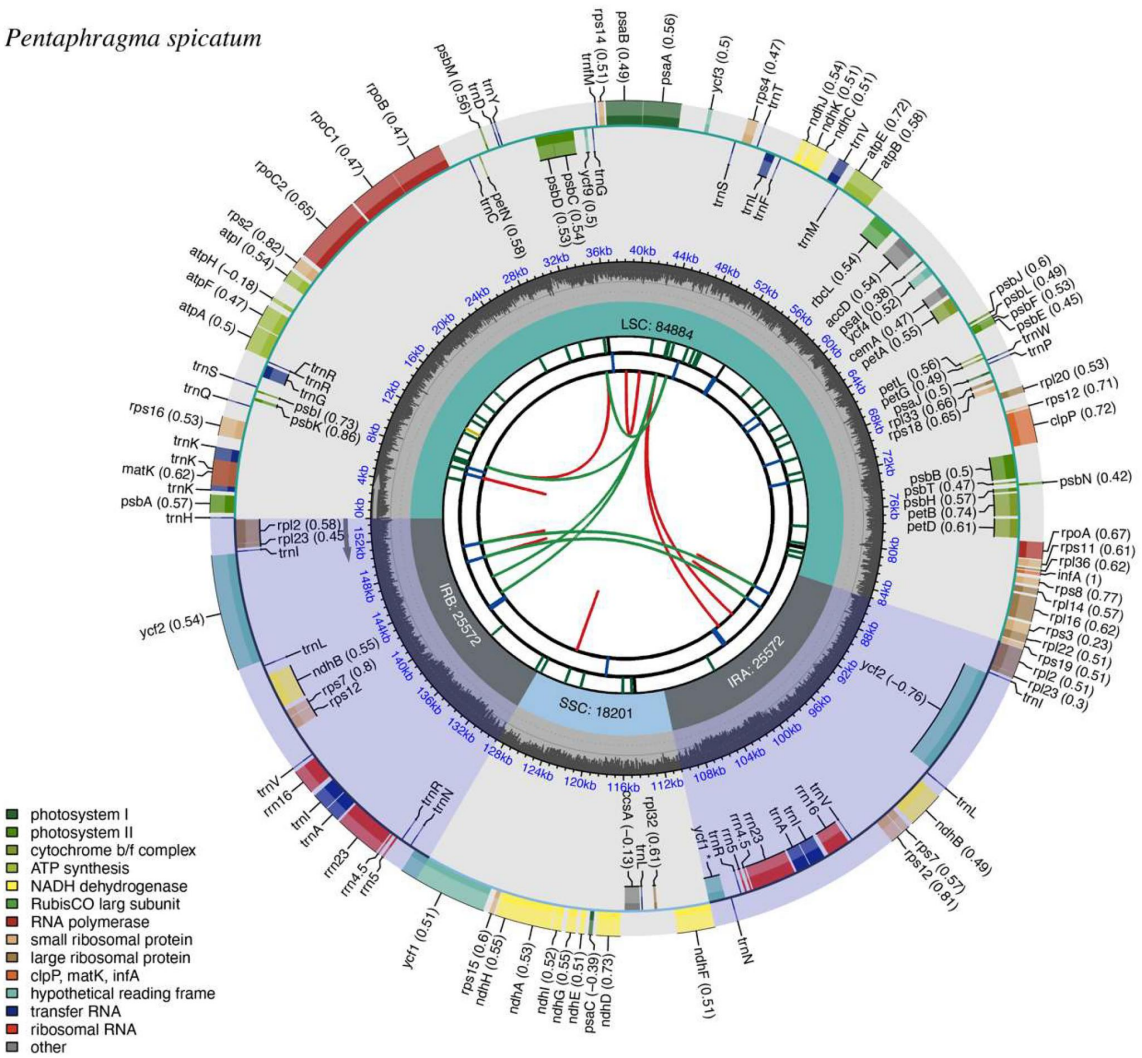
Schematic map of overall features of the *P. spicatum* chloroplast genome (genes drawn outside the outer circle are transcribed clockwise, and those inside are transcribed counter-clockwise. Genes belonging to different functional groups are color-coded. The different colored legends in the bottom left corner indicate genes with different functions. The dark grey inner circle indicates the GC content of the chloroplast genome and the presence of nodes in the LSC, SSC, IR regions).

The complete chloroplast genome of *P. spicatum* was composed of 154,229 base pairs (bp) and consists of a large single-copy region of 84,884 bp, a small single-copy region of 18,201 bp, and two inverted repeat regions of 25,572 bp, with an average depth of 1253.32 X (Figure S1). The overall GC content is 37.71%. The plastome contains a total of 129 genes, including 87 protein-coding genes (PCGs), 34 tRNAs, and 8 ribosomal RNAs (rRNAs). Furthermore, 16 genes in the chloroplast genome of *P. spicatum* contained introns. Among them, *rps*16, *atp*F, *rpo*C1, *pet*B, *pet*D, *rpl*16, *rpl*2, *ndh*A, *ndh*B, *trn*K-UUU, *trn*W-CCA, *trn*L-UAA, *trn*V-UAC, *trn*E-UUC, and *trn*A-UGC contained a single intron, whereas *clp*P had two introns. Additionally, *rps*12 had three and two exons located on the inverted repeats, indicating that *rps*12 exhibited trans-splicing (Figure S2). Consensus phylogenetic tree reconstructed by maximum likelihood (ML) and Bayesian inference (BI) analysis based on 56 protein-coding sequences (CDS) of 26 species, with *Lithospermum erythrorhizon*, *Trigonotis peduncularis*, and *Cordia dichotoma* as outgroups (Figure 2). The phylogenetic analysis suggests that Pentaphragmataceae is sister to Campanulaceae and Rousseaceae. The analysis further reveals a closer relationship between Rousseaceae and Campanulaceae than between Rousseaceae and the Pentaphragmataceae family.

## Discussion and conclusion

**Figure 3.**
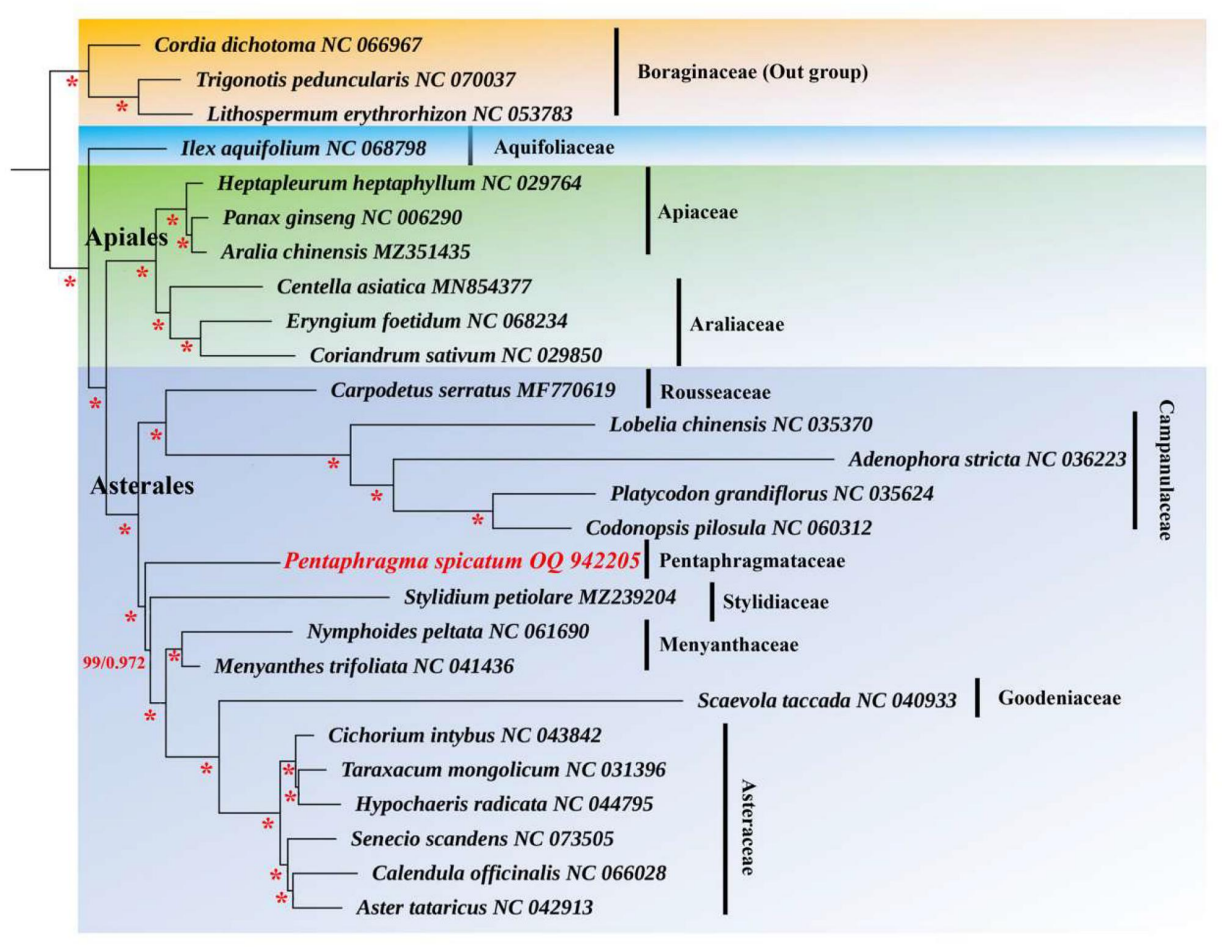
Consensus phylogenetic tree reconstructed by maximum likelihood (ML) and Bayesian inference (BI) analysis based on 56 protein-coding sequences (CDS) of 26 species, with *Lithospermum erythrorhizon*, *Trigonotis peduncularis*, and *Cordia dichotoma* as outgroups. Numbers near the branches are bootstrap support (BS) percentages obtained from maximum likelihood inference and posterior probabilities (PP) obtained from Bayesian analysis (BS/PP). those nodes with BS = 100%, PP = 1.00 were shown with asterisks. The following sequences were used: *Cordia dichotoma* NC_066967, *adenophora stricta* NC_036223 (Kyeong-Sik et al. 2017), *aralia chinensis* MZ351435, *aster tataricus* NC_042913 (Shen et al. 2018), *calendula officinalis* NC_066028, *carpodetus serratus* MF770619 (Knox 2014), *centella asiatica* MN854377, *cichorium intybus* NC_043842 (Yang et al. 2019), *Codonopsis pilosula* NC_060312, *Cordia dichotoma* NC_066967, *Coriandrum sativum* NC_029850, *Eryngium foetidum* NC_068234, *Heptapleurum heptaphyllum* NC_029764 (Zong et al. 2016), *Hypochaeris radicata* NC_044795, *Ilex aquifolium* NC_068798, *Lithospermum erythrorhizon* NC_053783, *Lobelia chinensis* NC_035370, *Menyanthes trifoliata* NC_041436, *Nymphoides peltate* NC_061690, *Panax ginseng* NC_006290, *Pentaphragma spicatum* OQ942205, *Platycodon grandifloras* NC_035642, *Scaevola taccada* NC_040933, *Senecio scandens* NC_073505, *Stylidium petiolare* MZ239204, *Taraxacum mongolicum* NC_031396, *Trigonotis peduncularis* NC_070037. The species newly sequenced in this study is shown in red font.

Previous studies revealed that the removal of *Pentaphragma* from Campanulaceae is supported by the sistership of Campanulaceae and Rousseaceae (*Pentaphragma* was not clustered in the Campanulaceae clade) based on three nucleotide sequence datasets (the chloroplast genes *atpB*, *ndhF*, and *rbcL*) in a phylogenetic analysis (Lundberg and Bremer 2003). Based on our results, it can be inferred that *P. spicatum* is not phylogenetically associated with Campanulaceae. Our results also support the classification of *Pentaphragma* as a component of the Pentaphragmataceae (Figure 3). The recently published chloroplast genome of *P. spicatum* will serve to augment and consolidate the present genome data. Thereby facilitating the refinement and integration of the existing genome data of Asterales. Furthermore, this will provide significant insights into the phylogenetic relationship within Campanulaceae.

In conclusion, our study provides further support for the taxonomic classification of *Pentaphragma* outside of the Campanulaceae family. Our findings reinforce the placement of *Pentaphragma* within the Pentaphragmataceae family, as opposed to its previous association with Campanulaceae. The newly published chloroplast genome of *P. spicatum* not only enriches our understanding of this particular species but also contributes to the broader efforts in enhancing and consolidating genome data within the Asterales order. This development holds the potential to refine and integrate existing genome datasets, ultimately shedding light on the intricate phylogenetic relationships within the Campanulaceae family. As we continue to explore the genetic diversity and relationships among plant species, studies like ours play a pivotal role in advancing our comprehension of botanical taxonomy and evolution.

## Supplementary Material

Supplemental Material

## Data Availability

The genome sequence data that support the findings of this study are openly available in GenBank of NCBI at [https://www.ncbi.nlm.nih.gov] (https://www.ncbi.nlm.nih.gov/) under accession no. OQ942205. The associated BioProject, SRA, and Bio-Sample numbers are PRJNA983960, SRR24928157, and SAMN35741070 respectively.
