# Supplementary material for: The first complete chloroplast genome sequence of Pentaphragma spicatum Merr. (Pentaphragmataceae) and phylogenetic analysis
Source: Mitochondrial DNA B Resour. 2023 Dec 12;8(12):1368–72. doi: 10.1080/23802359.2023.2290339 (PMC10776070; doi:10.1080/23802359.2023.2290339)
Supplement: Supplemental Material [file TMDN_A_2290339_SM7372.docx]

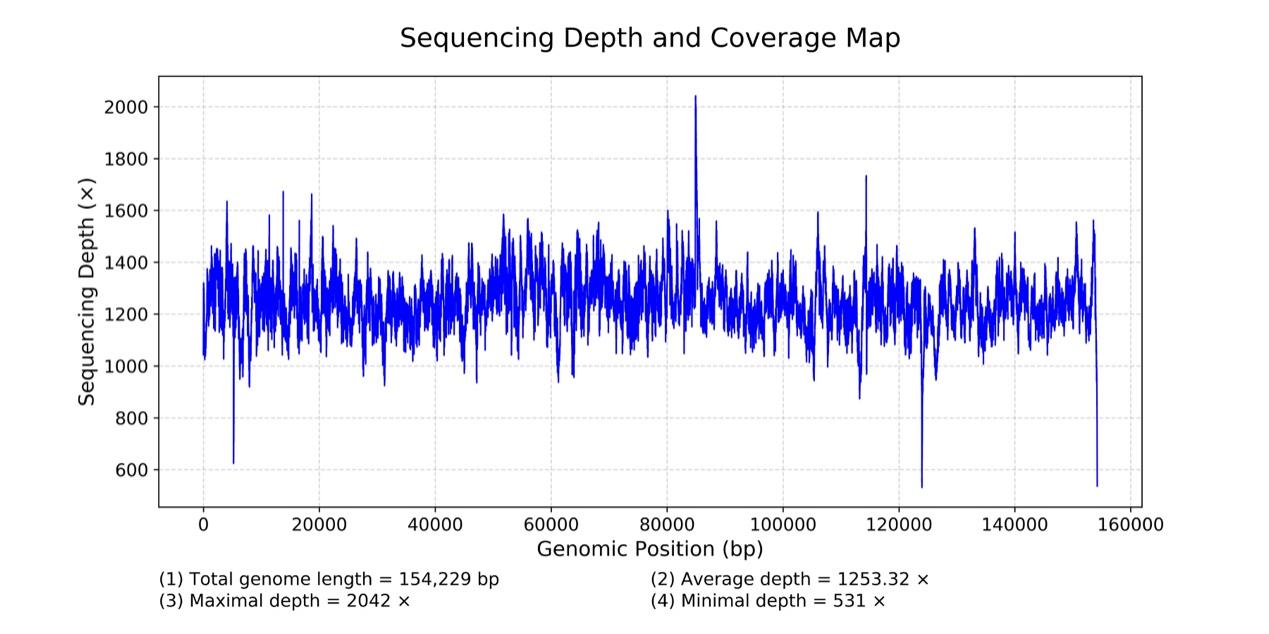


**Supplementary Figure S1**. Read mapping depth of the plastome sequence. Clean read

mapping depth is presented with blue bars. X and Y axis present nucleotide position of

plastome and read mapping depth, respectively. The minimum and average mapping depth are 531× and 1253.32×, respectively.


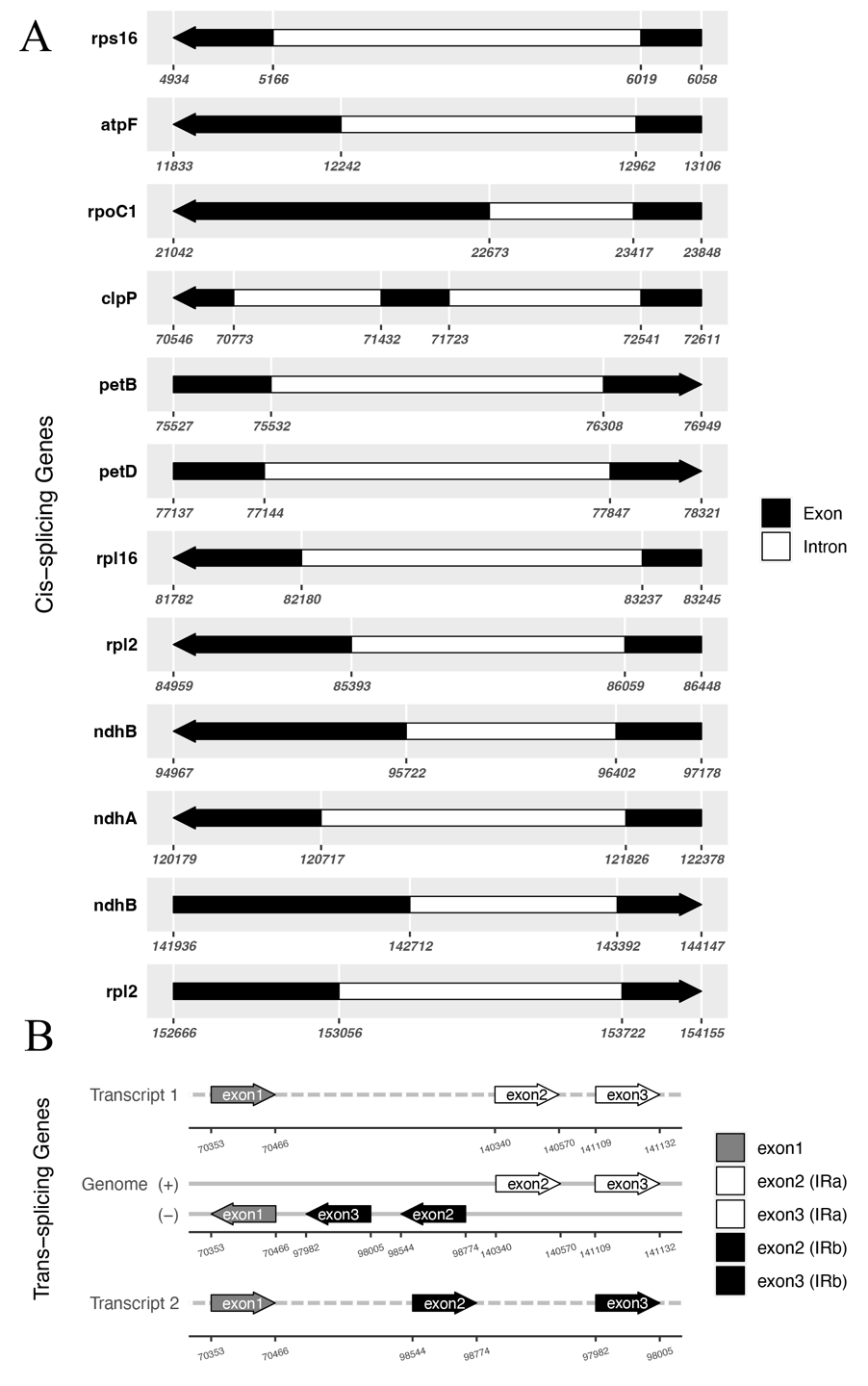


**Supplementary Figure S2**. Schematic map of the cis (A) and trans (B) splicing genes in the chloroplast genome of *Pentaphragma spicatum*.
